# Procedures for central auditory processing screening in schoolchildren^☆^

**DOI:** 10.1016/j.bjorl.2018.02.004

**Published:** 2018-03-22

**Authors:** Nádia Giulian de Carvalho, Thalita Ubiali, Maria Isabel Ramos do Amaral, Maria Francisca Colella-Santos

**Affiliations:** a Universidade Estadual de Campinas (UNICAMP), Faculdade de Ciências Médicas, Centro de Investigação em Pediatria (CIPED), Campinas, SP, Brazil; b Universidade Estadual de Campinas (UNICAMP), Faculdade de Ciências Médicas, Centro de Investigação em Pediatria (CIPED), Departamento de Desenvolvimento Humano e Reabilitação, Campinas, SP, Brazil

**Keywords:** Hearing, Hearing tests, Auditory perception, Child, Schoolchildren, Audição, Testes auditivos, Percepção auditiva, Criança, Estudantes

## Abstract

**Introduction:**

Central auditory processing screening in schoolchildren has led to debates in literature, both regarding the protocol to be used and the importance of actions aimed at prevention and promotion of auditory health. Defining effective screening procedures for central auditory processing is a challenge in Audiology.

**Objective:**

This study aimed to analyze the scientific research on central auditory processing screening and discuss the effectiveness of the procedures utilized.

**Methods:**

A search was performed in the SciELO and PUBMed databases by two researchers. The descriptors used in Portuguese and English were: auditory processing, screening, hearing, auditory perception, children, auditory tests and their respective terms in Portuguese. Inclusion criteria: original articles involving schoolchildren, auditory screening of central auditory skills and articles in Portuguese or English. Exclusion criteria: studies with adult and/or neonatal populations, peripheral auditory screening only, and duplicate articles. After applying the described criteria, 11 articles were included.

**Results:**

At the international level, central auditory processing screening methods used were: screening test for auditory processing disorder and its revised version, screening test for auditory processing, scale of auditory behaviors, children's auditory performance scale and Feather Squadron. In the Brazilian scenario, the procedures used were the simplified auditory processing assessment and Zaidan's battery of tests.

**Conclusion:**

At the international level, the screening test for auditory processing and Feather Squadron batteries stand out as the most comprehensive evaluation of hearing skills. At the national level, there is a paucity of studies that use methods evaluating more than four skills, and are normalized by age group. The use of simplified auditory processing assessment and questionnaires can be complementary in the search for an easy access and low-cost alternative in the auditory screening of Brazilian schoolchildren. Interactive tools should be proposed, that allow the selection of as many hearing skills as possible, validated by comparison with the battery of tests used in the diagnosis.

## Resumo

**Introdução:**

A triagem do processamento auditivo central em escolares tem suscitado discussões na literatura, tanto em relação ao protocolo a ser usado quanto à importância de ações voltadas para a prevenção e promoção da saúde auditiva. Um desafio na audiologia é definir procedimentos eficazes na triagem do processamento auditivo central.

**Objetivo:**

Analisar científicas que aplicaram triagem do processamento auditivo central e discutir a eficácia dos procedimentos usados.

**Método:**

Foi feita busca nas bases de dados SciELO e PUBMed por duas pesquisadoras. Os descritores usados em português e inglês foram: processamento auditivo, triagem, audição, percepção auditiva, crianças, testes auditivos. Critérios de inclusão: artigos originais que envolveram escolares, triagem auditiva das habilidades auditivas centrais e artigos em português ou inglês. Critérios de exclusão: pesquisas com a população adulta e/ou neonatal, apenas triagem auditiva periférica e artigos duplicados. Após aplicação dos critérios descritos foram incluídos 11 artigos.

**Resultados:**

No âmbito internacional, os métodos de triagem do processamento auditivo central foram o uso do *Screening Test for Auditory Processing Disorder* e sua versão revisada, *Screening Test for Auditory Processing*, os *checklists Scale of Auditory Behaviors* e *Children's Auditory Performance Scale* e o *Feather Squadron*. No cenário brasileiro, os procedimentos foram a avaliação simplificada do processamento auditivo e a bateria de Zaidan.

**Conclusão:**

No âmbito internacional, as baterias Screening Test for Auditory Processing e Feather Squadron se destacam pela avaliação mais completa das habilidades auditivas. Em âmbito nacional, há escassez de estudos que usem métodos que avaliem mais de quatro habilidades, com normatização para a faixa etária. A aplicação da avaliação simplificada do processamento auditivo e questionários pode se complementar na busca de uma opção de fácil acesso e baixo custo na triagem auditiva escolar brasileira. Devem ser propostas ferramentas interativas, que possibilite triar o maior número possível de habilidades auditivas, validada por meio da comparação com a bateria de testes usados no diagnóstico.

## Introduction

Auditory screening in school-aged children has been extensively studied in the literature, both regarding the protocol to be used and the importance of actions aimed at prevention and promotion of auditory health, since this age group is undergoing oral and written language development and auditory changes may interfere with the learning process. Recent studies indicate that the incidence of peripheral auditory alterations varies from 14% to 63.4%, depending on the evaluation procedure used.^1^, ^2^

It is known that peripheral auditory dysfunction and/or a history of secretory otitis media in the first five years of life can result in immaturity in the development of auditory pathways and central auditory abilities.^3^, ^4^ Auditory screening procedures are often used in the school environment to evaluate the function of the peripheral auditory system. Among the most frequently used methods, immittance screening is used to assess the middle ear functions^2^, ^5^, ^6^ and otoacoustic emission measures are used to assess cochlear functional integrity.^7^, ^8^

Listening to and understanding auditory information involves greater complexity central to the auditory periphery, since it involves the appropriate transmission of nerve impulses to the cochlear nuclei the, thalamus and auditory cortex. These auditory stations are responsible for sound localization and lateralization, auditory discrimination, auditory recognition, temporal aspects of hearing, auditory performance with competing acoustic signals, and auditory performance in unfavorable acoustic situations.^9^ Poor performance in one or more of these areas results in difficulty in processing auditory information in the central auditory system and is called Central Auditory Processing Disorder (CAPD).

The literature contains relevant discussions about the nature of the CAPD and its direct or indirect association with higher cognitive functions, such as attention, memory and language.^10^, ^11^, ^12^ Many investigators consider CAPD as a diagnostic entity identified in ICD-10 as diseases of the ear (H93.25), confirming the physiological nature (acquired or congenital) of this disorder. When children are diagnosed early enough, they can receive adequate support at school and therapy by qualified professionals, facilitating their development.^12^

The similarity of signs and behaviors in CAPD with other conditions such as Attention Deficit Hyperactivity Disorder (ADHD) and Attention Deficit Disorder (ADD) are confounding factors for the diagnosis and generate questions. However, although some symptoms are common in different clinical pictures, CAPD has signs and symptoms that are specifically related to auditory deficit, such as difficulty in understanding language spoken in noisy environments, when speech is quickly presented; difficulty with words of similar sounds and difficulty following complex auditory commands. Although these characteristic signs can be detected from the clinical history and symptom assessment through validated questionnaires,^13^ it is the consensus that the diagnosis of CAPD cannot be made based only on such questionnaire data, but more appropriately from an efficient battery of tests.^12^

With schoolchildren, care always must be taken to obtain a global speech-language assessment of the child and, whenever possible, a multiprofessional one, since CAPD might be an associated comorbidity with other clinical alterations, increasing diagnostic difficulties especially with respect to reading and writing performance.^14^, ^15^ Understanding the neural mechanisms through which sounds travel to the brain is necessary for aiding speech therapy and facilitating early diagnosis and effective intervention.^13^

Defining effective screening procedures to assess central auditory processing is a challenge for the field of Audiology. Since 1986, researchers have been searching for screening techniques, either through batteries of tests and/or questionnaires; however, there is no consensus regarding the most efficient protocol to be used for central auditory processing screening. Auditory screening should be a simple and rapid procedure that can be applied to many individuals, aiming at the early identification of those with a high probability of having a specific problem and, based on that identification, mandate a full evaluation.^16^, ^17^ The consequences of the lack of an effective standardized tool aimed at screening hearing skills vary from the lack of dissemination of knowledge regarding CAPD in the school environment, to the inability to obtain an epidemiological survey regarding CAPD diagnosis, especially in children with academic difficulties.^18^

Studies have shown that children with academic difficulties had worse speech perception in silence and in noise^19^, ^20^; therefore, rapid and effective screening procedures are required for children who may be at risk for CAPD. The aim of the present investigation was to analyze the studies that applied central auditory processing screening in schoolchildren, and to discuss the effectiveness of the procedures used.

## Methods

A review of the literature independently was performed by two researchers through an electronic search in the SciELO and PUBMed databases in the month of May of 2017. The research was carried out by crossing the following descriptors and their corresponding terms in English: *processamento auditivo* (auditory processing), *triagem* (screening), *audição* (hearing), *percepção auditiva* (auditory perception), *crianças* (children) and *testes auditivos* (hearing tests). All identified articles were included, according to the inclusion and exclusion criteria, regardless of the year of publication. The titles of the articles found in both databases were transported into an Excel spreadsheet for the exclusion of articles in duplicate during the matches made during the search.

Inclusion criteria: original articles involving schoolchildren, auditory screening with procedures focused on the evaluation of central auditory skills and articles in Portuguese and/or English languages.

Exclusion criteria: studies carried out with adult and/or neonatal populations, peripheral auditory screening only and duplicated articles.

**Figure 1:**
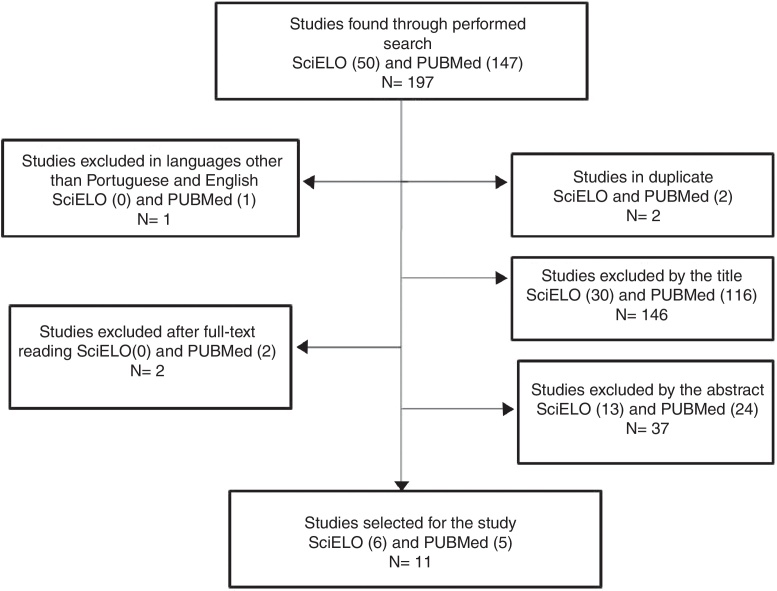
Explanatory diagram on the article selection process.

A total of 197 articles were identified, 50 in the SciELO database and 147 in the PUBMed database. After applying the exclusion criteria, 11 articles met the criteria and were included in the review. Fig. 1 illustrates the selection process.

## Results

The articles found in the SciELO and PUBMed databases, according to the criteria described in this review, were published between 1998 and 2016, as shown in Table 1.

**Table 1:** Overall data of selected articles, considering year of publication, authors, titles and databases.

N.	Year	Authors	Title	Database
1	1998	Amos and Humes	SCAN Test-Retest Reliability for First- and Third-Grade Children	PubMed
2	2006	Simon and Rossi	Auditory processing screening in school children from 8 to 10 years old	Scielo
3	2007	Lucas et al.	Scan: performance profile of children with seven to eight years	Scielo/Pubmed
4	2008	Rodrigues et al.	Auditory processing screening test (SCAN) performance profile in seven and eight year-old children living in Cuiabá	Scielo
5	2009	Colella-Santos et al.	Auditory assessment in the school-age children	Scielo
6	2012	Etges et al.	Acoustic immitance and auditory processing screening findings in school children	Scielo
7	2012	Toscano and Anastasio	Auditory abilities and acoustic immittance measures in children from 4 to 6 years old	Scielo
8	2013	Nunes et al.	Scale of Auditory Behaviors and auditory behavior tests for auditory processing assessment in Portuguese children	Scielo/Pubmed
9	2013	Yathiraj and Maggu	Screening Test for Auditory Processing (STAP): A Preliminary Report	PubMed
10	2016	Ahmmed and Ahmmed	Setting appropriate pass or fail cut-off criteria for tests to reflect real life listening difficulties in children with suspected auditory processing disorder	PubMed
11	2016	Barker and Purdy	An initial investigation into the validity of a computer-based auditory processing assessment (Feather Squadron)	PubMed

Table 2 shows the details of the analyzed studies regarding the sample characterization; screening method used and the main notes on the results and conclusions.

**Table 2:** Details of the reviewed studies.

N°	Sample	Screening	Results	Conclusion
1	Age: 6–9 years; G1: 25 children in First Grade; G2: 22 children in Third Grade; Native language: English.	SCAN (Keith, 1986): Filtered Speech, Speech in Noise, Competing (Dichotic) Words.	The retest with SCAN between 6 and 7 weeks showed significant improvement in the Filtered Speech and Competing Words tests. Only the Speech in Noise test showed no difference.	The second SCAN administration may provide a better estimate of the best performance of an individual child. The lack of standardization of the second score (retest) confounds a simple interpretation of such scores.

2	Age: 8–10 years; *n* = 106 students in the 2nd and 3rd Grades. After audiometry and the inclusion and exclusion criteria were applied, 57 individuals with typical development were evaluated, 33 females and 24 males; Native language: Brazilian Portuguese.	Zaidan's Auditory Processing Screening Battery (2001): Filtered Speech, Speech in Noise, Competing Words.	Filtered speech: statistically significant difference in the performance of individuals aged 8, 9 and 10 years. Speech in Noise and Competing Words: differences were not statistically significant. There was a statistically significant difference in the Total Battery, which is the sum of the correct answers in each of the subtests (Filtered Speech, Speech in Noise and Competing Words).	There was a statistically significant difference in the combined analysis of the performance of the individuals in the three age groups, considering the total auditory processing screening battery, that is, the results improved as the age increased.

3	*n* = 40 students, with no auditory or phonological complaints; Group 1 (*n* = 20; age: 7 years), Group 2 (*n* = 20; age: 8 years). Native language: Brazilian Portuguese.	Zaidan's Auditory Processing Screening Battery (2001): Filtered Speech, Speech in Noise, Competing Words.	The mean score for children aged 7 years in the Filtered Speech, Speech in Noise and Competitive Words tests was 33.35; 32.5 and 71.8, respectively; In the 8-year-old children it was: 33.5; 34.5 and 79.9.	Differences in findings compared to other studies demonstrate the need to evaluate a larger number of children from different geographic regions.

4	*n* = 215 children, with no auditory or phonological complaints; G1: 109 children aged 7 years; G2: 106 children aged 8 years; Native language: Brazilian Portuguese.	Zaidan's Auditory Processing Screening Battery (2001): Filtered Speech, Speech in Noise, Competing Words.	The mean scores and standard deviation in the Filtered Speech, Speech in Noise and Competing Words tests in children aged 7 years were, respectively, 24.4 ± 5.1; 33.4 ± 3.4 and 76.5 ± 9.7 points, and in children aged 8 years, they were, respectively, 24.0 ± 4.8; 34.0 ± 3.0 and 77.5 ± 10.8 points.	The scores of this study cannot be generalized to normal values for all Brazilian children due to regional variability.
5	Age: 5–10 years; *n* = 287 children; G1: children aged 5–6 years; G2: children aged 7–8 years; G3: children aged 9–10 years; Native language: Brazilian Portuguese.	Simplified Auditory processing assessment (ASPA): Sound Localization (SL), memory for verbal sounds (MSSV) and nonverbal sounds in sequence (MSSNV).	The schoolchildren of this study had more difficulty in memorizing sequences of sounds or temporal order than locating the sound source.	A total of 56% of the students passed the screening. Regarding the groups studied, Groups I and II had a larger number of children who failed the auditory screening, considering both immitance and auditory processing tests.
6	Age: 7–10 years; *n* = 130 students from 1st to 4th grades; Native language: Brazilian Portuguese.	Simplified Auditory processing assessment (ASPA): sound localization tests, memory for verbal sounds (MSSV) and nonverbal sounds in sequence (MSSNV).	A total of 76.15% of the children passed the ASPA. Moreover, it was observed that the test in which the students showed the worst performance was the memory for verbal sounds in sequence. 12.3% of the students failed the immittance screening and ASPA test.	Most of the subjects passed the ASPA test, with a higher frequency of correct answers in the sound localization test. There was no statistical association between the immittance screening result and the ASPA result.

7	Age: 4–6 years; *n* = 61 children; Native language: Brazilian Portuguese.	Simplified Auditory Processing Assessment (ASPA): sound localization tests, memory for verbal sounds (MSSV) and nonverbal sounds in sequence (MSSNV).	There was an alteration in at least one of the auditory skills investigated in 24.6% of the children.	Younger children showed a greater occurrence of alterations in auditory skill tests and acoustic immitance measures.

8	Age: 10–13 years; *n* = 51 Portuguese children with normal peripheral hearing; Native language: European Portuguese.	Scale of Auditory Behaviors (SAB) questionnaire adapted to European Portuguese was applied to the parents. The children were submitted to the Sound Localization, Memory for verbal sounds and Nonverbal Sounds in Sequence, Speech in Noise, Dichotic Digits test, Harmonic Pattern Dichotic Digits Test, Standard duration test and Gaps-In-Noise test.	A significant correlation was observed between the questionnaire score and the behavioral test results, with highest of them being observed in the tests related to temporal processing.	There was a correlation between the SAB score and the results obtained in the behavioral auditory tests in Portuguese children, suggesting that this questionnaire can be used in auditory processing screening.

9	Age: 8–13 years; *n* = 400 children with no hearing or language complaints; G1 (8–9 years, *n* = 82), G2 (9–10 years, *n* = 77), G3 (10–11 years, *n* = 78), G4 (11–12 years, *n* = 82), G5 (12–13 years, *n* = 81); English speakers.	Screening Test for Auditory Processing (STAP) divided into four subtests: (1) Speech perception in noise; (2) Dichotic consonant-vowel; (3) Gap detection; (4) Auditory Memory.	It was determined that 16% of the children were at risk for CAPD at one or more STAP subtests. Among these 16%, the auditory memory test was most often affected (73.4%), followed by binaural integration (65.6%), auditory separation/closure (59.4%) and temporal resolution (53.1%).	The STAP is able to detect three different mechanisms related to auditory processing (binaural integration, temporal resolution and speech perception in noise with auditory memory). The study also indicates that the number of children at risk for each of the different auditory processes varies.
10	Age: 6–11 years; *n* = 109 children with hearing complaints despite normal peripheral hearing; English speakers.	SCAN-C (Keith, 2000); IMAP (Moore et al., 2010); CHAPS questionnaire for teachers (Smoski et al., 1998).	There was a correlation between the tests and the CHAPS questionnaire.	Of the different CHAPS domains, only the Ideal CHAPS, Auditory Memory, and Attention were correlated with the TPAC.
11	G1 = 847 children aged 5–13 years, with normal peripheral hearing and no language or learning complaints; G2 = 46 children aged 5–14 years, who were reevaluated after 7 days (Feather Squadron and conventional evaluation); English speakers.	Feather Squadron: Lateralization and detection, Auditory memory, Temporal resolution, Dichotic listening, Figure-Ground and Speech in Noise.	A significant correlation was observed between the results of most auditory skills assessed with the Feather Squadron and the traditional auditory processing evaluation test.	The Feather Squadron test battery is a time-efficient, feasible, and reliable approach for auditory processing screening in school-aged children.

## Discussion

The basis of the discussion of the study carried out by Amos and Humes^21^ in 1998 is the use of the Screening Test for Auditory Processing Disorder (SCAN), which contains three subtests: Filtered Speech, Speech in Noise and Competing Words, as a screening method. The SCAN battery was standardized for ages 3–11 years to be widely used by audiologists in the United States, aiming to detect the possible causes of poor school performance in children rapidly, uniformly, and in a standardized manner.^22^ However, according to Amos and Humes, when the battery was applied between six and seven weeks after the first evaluation in 6–9 year old schoolchildren, an improvement was observed in the performance of children in the Filtered Speech and Competing Words tests, but not for the Speech in Noise test. Therefore, the interpretation of the screening findings at a second test application was questioned. These and other factors suggested a need for review and normalization of the SCAN.^22^

The battery was revised and expanded by adding competing sentences and modifying instructions to make it more understandable to younger children. The Competing Words test was reviewed and revised for children aged 5–11 years.^22^ Since then, the search for new strategies and scientific evidence based on SCAN has been ongoing.^22^, ^23^, ^24^ The advantages of the SCAN, include rapidity, since it requires only 20 min, and its utility, since it can be administered in the school environment, initially using a portable tape player. However, the SCAN only screens the auditory skills of Closure, Figure-Ground and Binaural Integration. The literature points out that school-aged children with learning impairment also may have difficulties with Temporal Processing.^25^, ^26^ Therefore, children with alterations in these skills would not be adequately screened with the SCAN.

In 2012, Yathiraj and Maggu developed the Screening Test for Auditory Processing (STAP) and in 2013, they applied this screening battery to 400 schoolchildren aged 8–13 years.^27^ The STAP initially consisted of four subtests: Speech in Noise Perception, Consonant-Vowel Dichotic, Gap Detection, and Auditory Memory tests. The authors demonstrated that the Speech Perception in Noise and Auditory Memory subtests could be grouped into a single subtest because of the association between Auditory Memory and Speech Perception in Noise; thereafter, the STAP consisted of three subtests.^27^ STAP has the advantages of being quick, lasting approximately 12 min, being easy to administer using a notebook and headset, and it can be given in a quiet room at school. However, it is still not possible to determine whether the STAP performance will be confirmed by the diagnostic tests. According to the researchers, the next step in the diagnosis of children screened with STAP is to confirm the identified risks.^27^ After that occurs, the battery can be recommended for auditory screening.^16^, ^17^

The questionnaires, or the so-called “checklists”, have been important internationally as a means of screening central auditory processing.^13^, ^28^ In 2013, the Scale of Auditory Behaviors (SAB) questionnaire, adapted to European Portuguese, was applied to the parents of 51 children aged 10–13 years. There was a significant correlation between the questionnaire score and that of the behavioral tests, with the highest correlation observed in the tests related to temporal processing. Of the children with scores lower than 46 points, 94.4% had alterations in one or more central auditory processing tests, suggesting that SAB can be used as a screening tool for auditory processing.^13^

In 2016, Ahmmed and Ahmmed^28^ used the Children's Auditory Performance Scale (CHAPS) and SCAN-C as screening methods and observed a correlation of the questionnaire with seven CAP tests. The CHAPS has six domains of listening conditions: (1) listening in noise (environment with other competing stimuli, e.g., television and people talking); (2) situation of listening in silence; (3) situation of ideal listening (no distractions and communicative action occurs with visual contact), (4) multiple inputs (in addition to listening there are other forms of input, such as visual and tactile); (5) auditory memory; and (6) auditory attention. Of the different CHAPS domains, only CHAPS with the ideal listening situation, auditory memory and attention were correlated with the CAP test.

The use of questionnaires is a simple and inexpensive procedure that can add information to the screening of at-risk children. Studies suggest that when the checklist procedures and auditory tests are applied in a complementary way, there is a correlation with the diagnostic tests.^29^

The use of interactive and easy-access tools has gained importance as a screening method.^24^, ^30^ Barker and Purdy^24^ developed a computer program called “Feather Squadron” that evaluates five auditory processing mechanisms: sound localization, auditory pattern recognition, temporal aspects of hearing, dichotic listening with competing acoustic signals and auditory performance with degraded acoustic signal. The screening was applied to 945 students aged 5–14 years using IPad tablets. A significant correlation was found between the Feather Squadron scores and the results of traditional auditory processing assessment in most CAP skills assessed by the battery. The study stands out in the international scenario as it covers five of the six auditory processing skills recommended by the American Speech-Language-Hearing Association (ASHA).^9^ The test can be completed in 30 min, can be used for schoolchildren screening and the study confirms the findings in the diagnostic battery.

In Brazil, the CAP screening procedure most commonly used with schoolchildren is the Simplified Auditory Processing Assessment (ASPA),^31^ consisting of dichotic procedures (free field and with sound instruments) and includes the skills of sound localization and temporal order (memory for verbal and nonverbal sounds in sequence). In the present study survey, three of the 11 identified studies (27.27%) used this method, as shown in Table 2.

Colella-Santos and collaborators^6^ screened 287 schoolchildren aged 5–10 years using the ASPA. In this study, 44% of schoolchildren failed the auditory processing screening; memorizing sound sequences and performing temporal ordering were the skills most often impaired. Children aged 5–8 years showed higher failure rates.

Toscano and Anastasio evaluated schoolchildren aged 4–6 years using ASPA and 24.6% showed at least one altered skill. The authors also found that younger children had a higher rate of abnormalities in auditory skill tests and acoustic immittance measures.^32^

The prevalence of hearing skill alterations found in another study^33^ using the ASPA, were similar to that of the previous study. Of the 130 students between 7 and 10 years, 23.85% failed the screening, exhibiting worse performance in the memory for verbal sounds in sequence and better performance in sound localization.

For younger children, the ASPA has widespread applicability because it requires only a brief administration time and requires readily accessible materials,. However, performance on the test improves with advancing age until pre-adolescence. Since the battery does not evaluate auditory mechanisms, it is unable to identify schoolchildren with other auditory mechanism impairments. However, the ASPA can be a sensitive predictor of CAPDs, because when the assessed auditory mechanisms have matured, and when the skills are altered, the behavioral tests are very likely to be altered, since they include more complex tasks.^34^

Another CAP screening method used in Brazil is the Zaidan battery, which consists in the SCAN adapted to Portuguese.^35^ The battery was applied to children aged 6–11 years who had normal hearing levels and no speech, language or CAPD alterations; subsequently, it was evaluated in children with a clinical diagnosis of CAPD. The results were not conclusive due to the small number of children in the experimental group (*n* = 11) and the great variation in age in the group.^35^ However, the Zaidan screening battery was utilized in more recent studies in 2006, 2007 and 2008, that were included in this article, representing three of the 11 identified studies (27.27%).

In 2006, researchers applied the SCAN to 57 children aged 8–10 years using a portable stereo disk-player and two headphones.^18^ They observed an improvement in the screening test performance as age increased, when scores were considered in the total auditory processing screening battery, which is the sum of the correct answers in each of the subtests (Filtered Speech, Speech in Noise and Competing Words). There was a statistically significant difference in the combined analysis of the individuals’ performance for the three age groups, demonstrating the ability of the test to evaluate maturation of the central auditory nervous system.^18^

In 2007, the battery was applied to 40 children aged 7 and 8 years in the municipality of Bauru, state of São Paulo, using an audiometer coupled to the Compact Disc Player (CD) to establish a standard of normal for the SCAN test in central auditory processing evaluation, and to be able to compare the findings with those in Zaidan's study. However, differences in scores were observed. According to the researchers, it is necessary to execute a study with larger numbers of children and children from different geographic regions.^23^

In 2008, the Zaidan battery was again applied to schoolchildren aged 7 and 8 years old in the municipality of Cuiabá, state of Mato Grosso.^36^ The researchers analyzed the performance profile in an auditory processing screening test in 215 students, using a portable stereo CD player. However, according to the researchers, the score results of this battery showed statistically significant differences in the Filtered Speech and Competing Words subtests, compared to the previous study by Zaidan (2001). The authors believe that such differences require multicenter studies that consider the social, cultural and ethnic differences of the tested individuals.

These studies^18^, ^23^, ^36^ indicate that it is difficult to generalize about the standard of normal from the results obtained using the battery standardized by Zaidan. Another significant aspect of the Zaidan battery is that it does not evaluate temporal processing assessment mechanisms, as mentioned and discussed in relation to the use of the SCAN in its American version. So the use of the Zaidan battery as a screening method does not allow the early identification of children with temporal processing difficulties.

With the advances in technology, the utilization of Telehealth in audiology has become prominent as a means to improve access to auditory health services and quality of care.^37^ Currently, the use of teleaudiology in peripheral auditory screening in children is gaining traction.^30^ Concerns with peripheral hearing were described in most of the studies analyzed in this review, with emphasis on the study of tonal thresholds in the articles identified in Table 1, Table 2 from 1 to 4; study of middle ear conditions through Immittance measures in Studies 5 and 6, or the combined use of tonal threshold and Immittance measures in Studies 7 and 8. When peripheral auditory screening was not performed immediately before CAP screening, the criterion of performing the procedure within 72 h after CAP screening was considered, mainly to ensure middle ear function conditions (Study 11). It is known that mild to moderate hearing loss is common in schoolchildren and chronic otitis media is its main cause, affecting approximately 80% of children and resulting in at least one occurrence of temporary hearing loss during the year.^38^

The central auditory processing screening initiatives reviewed in this study are satisfactory with respect to being easy to apply and short duration. However, it is necessary to consider the reality of each geographic region with respect to feasibility; the use of audiometers, IPADs and/or computers may not be possible in some school environments.

The use of only one tool such as the ASPA or the questionnaire also does not address the complexity of CAP screening, since none of the methods can address, in a single battery, all the auditory mechanisms underlying the hearing skills recommended by ASHA.^9^ The use of ASPA and the questionnaires can complement each other in the search for an easy-access and low-cost alternative. Another challenge is to adapt adequate auditory screening to different age groups. In this review, the assessed age range varied from 4 to 14 years of age, through six combined or not combined screening possibilities.

Based on the analyzed studies, there is a need for technological resources that identify evaluation procedures that include the auditory skills in the audiological evaluation. The STAP and Feather Squadron have a more complete battery for CAP screening. The Feather Squadron was applied in the age group of 5–14 years, and divided the battery in two versions, for children aged 5–7 years and those older than 8 years. The STAP battery was applied only in schoolchildren older than 8 years, reflecting the maturation of CAP skills by that age. Considering the analyzed studies, it is clear that central auditory processing screening is an area yet to be explored.

New studies should be carried out that aim to identify a battery of procedures that screen as many auditory skills as possible, that can evaluate different age groups, and then be validated by comparison with the battery of tests used in the CAPD diagnosis.

## Conclusion

At the international level, two screening batteries can be highlighted for their most comprehensive assessment of hearing skills – STAP and Feather Squadron. At the national level, there is a shortage of studies that use central auditory processing screening methods that evaluate more than four central auditory processing skills. The association of questionnaires in schoolchildren screening practices, the use of ASPA for pre-school children and the development and study of new auditory screening proposals for older children are suggested, including procedures aimed at the evaluation of central auditory skills that can be applied in the school environment. Such procedures should be validated based on studies that confirm the schoolchildren's performance in central auditory processing screening by comparing it with the performance in the CAP behavioral assessment battery tests.

## Conflicts of interest

The authors declare no conflicts of interest.

## References

[1] Dadalto E.V., Nielsen C.S.C.B., Oliveira E.A.M., Taborda A. (2012). Prevalence of communication disorders in scholars of the municipal elementary school network of Vila Velha/ES. Rev CEFAC.

[2] Lindau T.A., Delecrode C.R., Cardoso A.C.V. (2013). Achados timpanométricos em um grupo de escolares. Rev CEFAC.

[3] Borges L.R., Paschoal J.R., Colella-Santos M.F. (2013). (Central) Auditory Processing: the impact of otitis media. Clinics.

[4] Bayat A., Farhadi M., Emamdjomeh H., Saki N., Mirmomeni G., Rahim F. (2017). Effect of conductive hearing loss on central auditory function. Braz J Otorhinolaryngol.

[5] Araújo S.A., Moura J.R., Camargo L.A., Alves W. (2002). Avaliação auditiva em escolares. Rev Bras Otorrinolaringol.

[6] Colella-Santos M.F., Bragato G.R., Martins P.M.F., Dias A.B. (2009). Triagem auditiva em escolares de 5 a 10 anos. Rev CEFAC.

[7] Farias V.V., Camboim E.D., Azevedo M.F., Marques L.R. (2012). Ocorrência de falhas na triagem auditiva em escolares. Rev CEFAC.

[8] Tamanin D., Ramos N., Dutra L.V., Bassanesi H.J.C. (2015). Triagem auditiva escolar: identificação de alterações auditivas em crianças do primeiro ano do ensino fundamental. Rev CEFAC.

[9] American Speech-Language-Hearing Association [Internet] (2005). http://www.asha.org/policy.

[10] Moore D.R., Ferguson M.A., Edmondson-Jones A.M., Ratib S., Riley A. (2010). Nature of auditory processing disorder in children. Pediatrics.

[11] Ahmmed A.U., Ahmmed A.A., Bath J.R., Ferguson M.A., Plack C.J., Moore D.R. (2014). Assessment of children with suspected auditory processing disorder: a factor analysis study. Ear Hear.

[12] Chermak G.D., Musiek F.E., Weihing J. (2017). Beyond controversies: the science behind central auditory processing disorder. Hear Rev.

[13] Nunes C.L., Pereira L.D., Carvalho G.S. (2013). Scale of Auditory Behaviors e testes auditivos comportamentais para avaliação do processamento auditivo em crianças falantes do português europeu. CoDAS.

[14] Carvalho N.G., Novelli C.V.L., Colella-Santos M.F. (2015). Fatores na infância e adolescência que podem influenciar o processamento auditivo: revisão sistemática. Rev CEFAC.

[15] Souza M.A., Passaglio N.J.S., Lemos S.M.A. (2016). Alterações de linguagem e processamento auditivo: revisão de literatura. Rev CEFAC.

[16] Engelman L., Ferreira M.I.D.C. (2009). Avaliação do processamento auditivo em crianças com dificuldades de aprendizagem. Rev Soc Bras Fonoaudiol.

[17] Northern J.L., Downs M.P., Northern J.L., Downs M.P. (2002). Hearing in children.

[18] Simon L.F., Rossi A.G. (2006). Triagem do processamento auditivo em escolares de 8 a 10 anos. Psicol Esc Educ.

[19] Anderson S., Chandrasekaran B., Yi H.G., Kraus N. (2010). Cortical-evoked potentials reflect speech-in-noise perception in children. Eur J Neurosci.

[20] Carvalho N.G., Novelli C.L., Colella-Santos M.F. (2017). Evaluation of speech in noise abilities in school children. Int J Pediatr Otorhinolaryngol.

[21] Amos N.E., Humes L.E. (1998). SCAN test–retest reliability for first-and third-grade children. J Speech Lang Hear Res.

[22] Keith R.W. (2000). Development and standardization of SCAN-C test for auditory processing disorders in children. J Am Acad Audiol.

[23] Lucas P.A., Zacare C.C., Alves F.O.C., Amantini R.C.B., Bevilacqua M.C., Zaidan E. (2007). Scan: perfil de desempenho em crianças de sete e oito anos. Pró-Fono R Atual Cient.

[24] Barker M.D., Purdy S.C. (2016). An initial investigation into the validity of a computer-based auditory processing assessment (Feather Squadron). Int J Audiol.

[25] Oliveira J.C., Murphy C.F.B., Schochat E. (2013). Processamento auditivo (central) em crianças com dislexia: avaliação comportamental e eletrofisiológica. CoDAS.

[26] Santos T.S., Mancini P.C., Sancio L.P., Castro A.R., Labanca L., Resende L.M. (2015). Achados da avaliação comportamental e eletrofisiológica do processamento auditivo. Audiol Commun Res.

[27] Yathiraj A., Maggu A.R. (2013). Screening test for auditory processing (STAP): a preliminary report. J Am Acad Audiol.

[28] Ahmmed A.U., Ahmmed A.A. (2016). Setting appropriate pass or fail cut-off criteria for tests to reflect real life listening difficulties in children with suspected auditory processing disorder. Int J Pediatr Otorhinolaryngol.

[29] Wilson W.J., Jackson A., Pender A., Rose C., Wilson J., Heine C. (2011). The CHAPS SIFTER, and TAPS-R as predictors of (C)AP skills and (C)APD. J Speech Lang Hear Res.

[30] Skarzyński P.H., Świerniak W., Piłka A., Skarzynska M.B., Wlodarczyk A.W., Kholmatov D. (2016). A hearing screening program for children in primary schools in Tajikistan: a telemedicine model. Med Sci Monit.

[31] Pereira L.D., Schochat E., Pereira L.D., Schochat E. (1997). Processamento auditivo central: manual de avaliação.

[32] Toscano R.D.G.P., Anastasio A.R.T. (2012). Habilidades auditivas e medidas da imitância acústica em crianças de 4 a 6 anos de idade. Rev CEFAC.

[33] Etges C.L., Reis M.C.P., Menegotto I.H., Sleifer P., Soldera C.L.C. (2012). Achados na triagem imitanciométrica e de processamento auditivo em escolares. Rev CEFAC.

[34] Vargas G.C., Ferreira M.I.D.C., Vidor D.C.G.M., Machado M.S. (2014). Avaliação simplificada e comportamental do processamento auditivo em escolares: estabelecendo relações. Rev CEFAC.

[35] Zaidan E. (2001).

[36] Rodrigues P.A.L., Sameshima K., Zaidan E. (2008). Perfil de desempenho em teste de triagem de processamento auditivo (SCAN) em crianças de sete e oito anos residentes em Cuiabá. Rev Soc Bras Fonoaudiol.

[37] Molini-Avejonas D.R., Rondon-Melo S., Amato C.A., Samelli A.G. (2015). A systematic review of the use of telehealth in speech, language and hearing sciences. J Telemed Telecare.

[38] Klausen O., Moller P., Holmefjord A., Reisaeter S., Asbjornsen A. (2000). Lasting effects of otitis media with effusion on language skills and listening performance. Acta Otolaryngol Suppl.

